# Longitudinal Development of Peripapillary Hyper‐Reflective Ovoid Masslike Structures Suggests a Novel Pathological Pathway in Multiple Sclerosis

**DOI:** 10.1002/ana.25782

**Published:** 2020-06-09

**Authors:** Axel Petzold, Danko Coric, Lisanne J. Balk, Steffen Hamann, Bernard M. J. Uitdehaag, Alastair K. Denniston, Pearse A. Keane, David P. Crabb

**Affiliations:** ^1^ Dutch Expertise Center for Neuro‐Ophthalmology and Multiple Sclerosis Center, Departments of Neurology and Ophthalmology Amsterdam University Medical Center Amsterdam The Netherlands; ^2^ Moorfields Eye Hospital and National Hospital for Neurology and Neurosurgery London United Kingdom; ^3^ University College London Queen Square Institute of Neurology London United Kingdom; ^4^ National Institute for Health Research Biomedical Research Centre at Moorfields Eye Hospital and University College London Institute of Ophthalmology London United Kingdom; ^5^ Multiple Sclerosis Center and Department of Neurology Amsterdam University Medical Center Amsterdam The Netherlands; ^6^ Department of Ophthalmology, Rigshospitalet University of Copenhagen Copenhagen Denmark; ^7^ Department of Ophthalmology University Hospitals Birmingham National Health Service Foundation Trust Birmingham United Kingdom; ^8^ Academic Unit of Ophthalmology Institute of Inflammation and Ageing, University of Birmingham Birmingham United Kingdom; ^9^ Optometry and Visual Sciences City, University of London London United Kingdom

## Abstract

**Objective:**

Peripapillary hyper‐reflective ovoid masslike structures (PHOMS) are a new spectral domain optical coherence tomography (OCT) finding.

**Methods:**

This prospective, longitudinal study included patients (n = 212) with multiple sclerosis (MS; n = 418 eyes), 59 healthy controls (HCs; n = 117 eyes), and 267 non‐MS disease controls (534 eyes). OCT and diffusion tensor imaging were used.

**Results:**

There were no PHOMS in HC eyes (0/117, 0%). The prevalence of PHOMS was significantly higher in patients with MS (34/212, *p* = 0.001) and MS eyes (45/418, *p* = 0.0002) when compared to HCs (0/59, 0/117). The inter‐rater agreement for PHOMS was 97.9% (kappa = 0.951). PHOMS were present in 16% of patients with relapsing–remitting, 16% of patients with progressive, and 12% of patients with secondary progressive disease course (2% of eyes). There was no relationship of PHOMS with age, disease duration, disease course, disability, or disease‐modifying treatments. The fractional anisotropy of the optic radiations was lower in patients without PHOMS (0.814) when compared to patients with PHOMS (0.845, *p* = 0.03). The majority of PHOMS remained stable, but increase in size and de novo development of PHOMS were also observed. In non‐MS disease controls, PHOMS were observed in intracranial hypertension (62%), optic disc drusen (47%), anomalous optic discs (44%), isolated optic neuritis (19%), and optic atrophy (12%).

**Interpretation:**

These data suggest that PHOMS are a novel finding in MS pathology. Future research is needed to determine whether development of PHOMS in MS is due to intermittently raised intracranial pressure or an otherwise impaired “glymphatic” outflow from eye to brain. **ANN NEUROL 2020;88:309–319.**

## Introduction

The optic disc has been of interest in multiple sclerosis (MS) since the original description of structural changes observed following optic neuritis.^1^, ^2^ Early postmortem histological observations of the optic disc were that “nerve‐fibers showed numerous spindle‐shaped swellings […]. Adhering to the nerve‐fibers were very numerous ovoid flattened nuclei […].”^3^ This was interpreted to represent “nutritive hyperplasia” supposedly of “increased activity of the protoplasm.”^3^

Longitudinal study of these ovoid structures has been challenging, because they are buried below the nerve fiber layer and due to the need for histology. With the introduction of retinal optical coherence tomography (OCT), it has become possible to study retinal structures in much more detail than previously possible.^4^ These ovoid structures have recently been termed peripapillary hyper‐reflective ovoid masslike structures (PHOMS) by an international consensus panel.^5^ Current evidence suggests that these PHOMS originate from axoplasmic stasis or congestion in the prelaminar optic nerve head.^6^ The occurrence of PHOMS in MS has not yet been studied, with studies almost exclusively focusing on quantitative assessment of the peripapillary retinal nerve fiber layer (pRNFL) from an OCT ring scan rather than qualitative assessment of optic disc volume scans.^4^, ^7^

In this longitudinal, prospective study, we investigated the occurrence and development of PHOMS and optic disc drusen (ODD) in patients with MS and healthy control subjects.^8^ We also studied how this novel observation related to non‐MS disease controls and MS‐specific clinical data and integrity of the visual pathways on brain magnetic resonance imaging (MRI).

## Subjects and Methods

This prospective study was approved by the ethics committee of the Amsterdam University Medical Center (protocol number 2010/336) and the scientific research committee (protocol number CWO/10‐25D). The study is in accordance with the Declaration of Helsinki. Written informed consent was obtained from all subjects prior to study inclusion.

### *Study Design and Patients*

#### *MS Disease Population*

All subjects were recruited at the Multiple Sclerosis Center at Amsterdam University Medical Center between March 2011 and August 2012. Patients were assessed at baseline and after 2 years.

Inclusion criteria were a diagnosis of MS according to the 2010 revision of the McDonald diagnostic criteria.^9^ Exclusion criteria were pregnancy, a relapse or a course of steroids in the past 4 weeks, a diagnosis of human immunodeficiency virus or other immunodeficiency, substance abuse in the past 5 years, or MRI findings that could interfere with evaluation. For healthy controls, additional exclusion criteria were any other neurological, ophthalmological, or psychiatric disease or a first‐ or second‐degree relative with a diagnosis of MS. Episodes of MS optic neuritis (MSON) were identified through patient history and confirmed clinically using a standard care protocol.^10^ Subjects with ODD were excluded as per OSCAR‐IB criteria.^11^ At baseline screening, a total of 53 eyes were excluded due to opacities in the visual pathways, abnormal retinal or optic disc findings, or other problems.^12^ To be consistent with previous publications on this cohort,^12^, ^13^ the disease course was classified into relapsing–remitting, secondary progressive, and primary progressive according to the Lublin and Reingold classification.^14^

#### *Non‐MS Disease Control Population*

The retrospective case note review was approved by the Research and Development Department of Moorfields Eye Hospital, London (protocol number ROAD17/030). The diagnostic groupings were optic atrophy, isolated optic neuritis, referrals for assessment of an incidentally detected anomalous optic disc, increased intracranial hypertension (IIH), ODD, medical retinal diseases, headaches, nonembolic transient visual field loss, and those who experienced entoptic phenomena.

The retrospective control population was added after the MS cohort study had finished. Therefore, only descriptive statistics were performed to illustrate the general distribution of PHOMS as may be encountered in clinic.

#### *OCT Protocol*

All OCT images were obtained by spectral‐domain OCT (Heidelberg Spectralis; Heidelberg Engineering, Heidelberg, Germany; software v1.1.6.3) with eye‐tracking function enabled for best accuracy.^15^ Data were collected from an optic disc volume scan (15° × 15°, 37 B‐scans), peripapillary ring scan (12°, 1 B‐scan), and macular volume scan (20° × 20°, 49 B‐scans). We could not include enhanced depth imaging (EDI), because this feature was added at a later stage to the software.

Automated segmentation was performed with the manufacturer's software (HEYEX v1.10.2.0, Viewing Module v6.9.5.0). All scans underwent a rigorous quality control check.^11^ Algorithm failures were corrected by hand. The pRNFL, macular ganglion cell inner plexiform layer (GCIPL), and macular inner nuclear layer thicknesses were exported for statistical analysis. All OCT terminology used follows consensus guideline recommendations.^16^ The ODD consortium definition of PHOMS for OCT was used based on consensus.^5^ We did not perform ultrasound.

### *MRI Protocol*

Structural MRI was performed on a 3T whole body system (SIGNA HDxt; GE Healthcare, Milwaukee, WI). The detailed acquisition parameters have been described previously as well as an example of the 3T MRI.^12^, ^13^ In brief, normalized gray and white matter volumes and lesion volumes were quantified automatically using k nearest neighbor classification with tissue type priors, and SIENAX (part of FMRIB Software Library 5.0.4, http://www.fmrib.ox.ac.uk/fsl). Lesion filling was applied to minimize the effect of lesions on atrophy measurements.

#### *External Validation*

External validation of the PHOMS rating was performed (S.H.). The inter‐rater agreement for rating of PHOMS has been investigated using Fleiss kappa statistics. The inter‐rater kappa for the 2 independent raters of PHOMS in the present study (A.P., S.H.) was 0.811 in the multirater study of the ODD consortium.^17^

#### *Statistical Analysis*

The statistical analyses were performed in SAS (v9.4). First, normality in measurements was tested graphically and using Shapiro–Wilk statistics. Nonparametric tests were used for non‐normal or skewed data and parametric tests for normally distributed data. Median (interquartile range) or mean ± standard deviation are shown. Differences between 2 groups were analyzed using the chi‐squared test for categorical variables, the 2‐tailed *t* test for parametric continuous variables, and the Mann–Whitney test for nonparametric continuous variables. General linear models were used for comparison of data from >2 groups. Correlation analyses were performed using Pearson *r* for normally distributed and Spearman rho for non‐Gaussian data. Bonferroni method was used to correct for multiple correlations. Differences for segmented retinal layer thickness data between groups was analyzed using generalized estimation equations as recommended^16^; these were adjusted for intrasubject intereye correlations and repeated measurements, and employed an exchangeable correlation structure. Inter‐rater agreement on rating of PHOMS was assessed using Cohen kappa. Missing data were handled as such and indicated in the footnotes to the tables. A *p* value of 0.05 was accepted as statistically significant.

### *Data Availability Statement*

Anonymized data will be shared upon request from qualified investigators.

## Results

The baseline data of the 227 patients with MS and 62 control subjects are summarized in Table 1. Patients with MS were only slightly older on average compared to the control subjects (*p* = 0.0105; 95% mean difference = 3.5 years, 95% confidence interval = 1 to 6 years).

**TABLE 1:** Subject Characteristics

	Controls	Patients	*p*
Subjects	62	227	
Eyes	117	418	
Gender, F:M	41:21	155:72	ns
Age, yr	50.6 (7.1)	54.1 (10.0)	0.01^a^
Disease duration, yr	n/a	20.4 (6.9)	
Follow‐up, mo	27.5 (2.9)	26.0 (2.7)	ns
EDSS	n/a	4.2 (1.7)	
EDSS progression	n/a	0.3 (0.7)	
Disease course	n/a	139 RR, 28 PP, 60 SP	
MSON	None	32 right, 30 left, 39 bilateral, 126 never	
DMT	n/a	IFN, 65; FTY, 4; AZT, 1; MTX, 3; 1, ATM‐027; NTZ, 10; GA, 15; teriflunomide, 1	
ODD	1 (2%)	1 (1%)	ns
PHOMS	0 (0%)	34 (16%) all MS, 21 (16%) RR, 6 (12%) SP, 7 (16%) PP	*<*0.001^b^

Mean (standard deviation) or n (%) is shown.

a The Bonferroni adjusted *p* value for multiple comparisons (n = 5) is 0.01.

b The statistical significance of this finding increases to *p* < 0.0001 if the number of eyes (45/373 vs 0/117) instead of the number of patients (as shown in the table) is taken for comparison.

AZT = azathioprine; DMT = disease‐modifying treatment; EDSS = Expanded Disability Status Scale; F = female; FTY = fingolimod; GA = glatiramer acetate; IFN = interferon beta 1a and 1b; M = male; MS = multiple sclerosis; MSON = multiple sclerosis optic neuritis; MTX = mitoxantrone; n/a = not applicable; ns = not significant; NTZ = natalizumab; ODD = optic disc drusen; PHOMS = peripapillary hyper‐reflective ovoid masslike structure; PP = primary progressive; RR = relapsing–remitting; SP = secondary progressive.

**FIGURE 1: (A):**
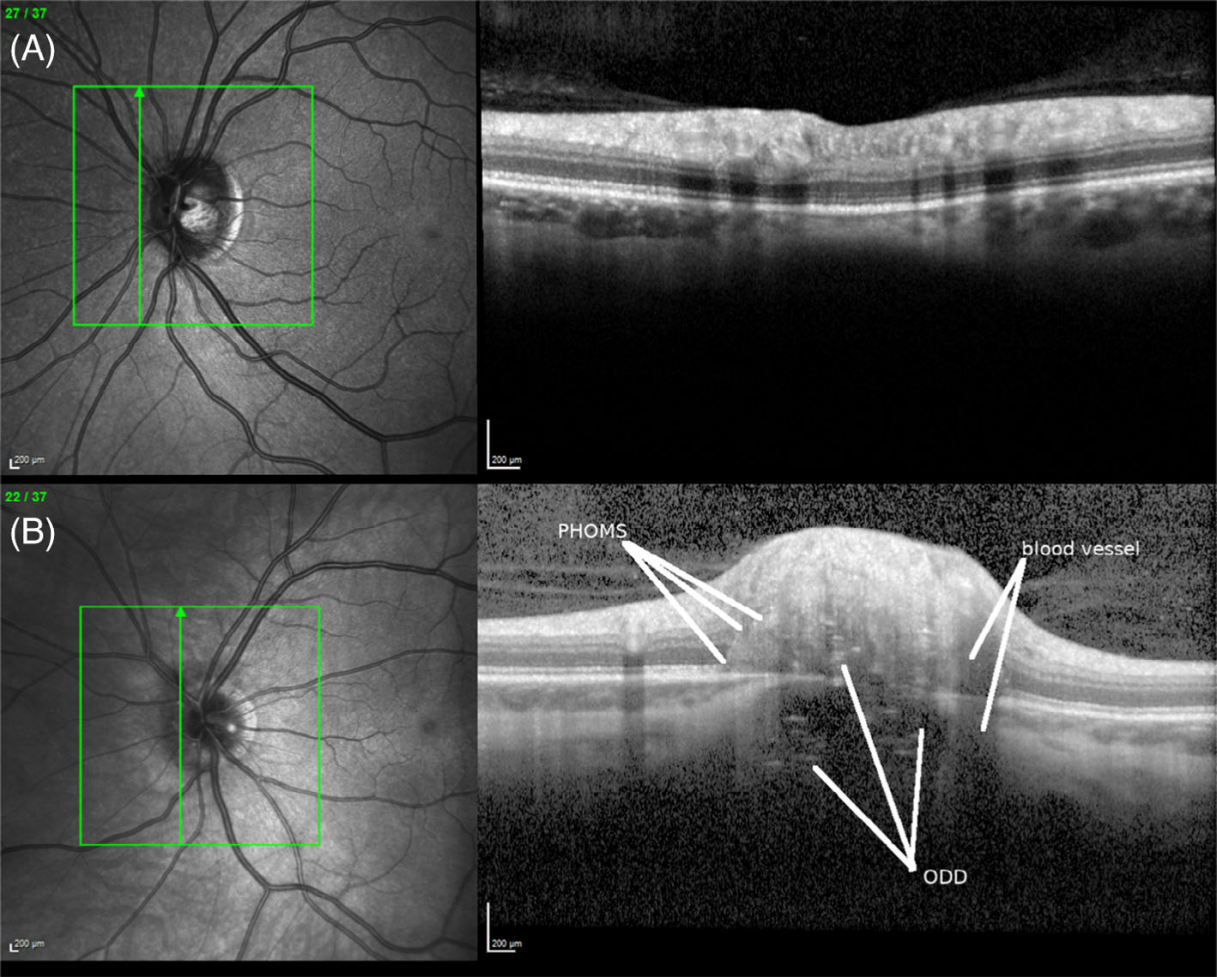
A normal optic disc. (B) An optic disc with peripapillary hyper‐reflective ovoid masslike structures (PHOMS) and optic disc drusen (ODD). The hyperintense PHOMS typically transverse several of the retinal layers, in this case from the retinal nerve fiber layer down to the basal membrane. The ODD are located above and below Bruch's membrane and impress as conglomerates of low‐intensity signal with intermingled hyperintense small horizontal lines. This appearance is very different from the vertical shadow cast by blood vessel artifacts. The confocal laser scanning ophthalmoscopy is shown on the left. The green lines indicate the location of the optical coherence tomographic B‐scan shown on the right. [Color figure can be viewed at www.annalsofneurology.org]

Figure 1 shows the appearance of a normal optic disc in comparison to an optic disc with PHOMS. The disc shown in Figure 1B represents 1 of the 2 cases where PHOMS did coexist with ODD. Because ODD were an exclusion criterion, these 2 cases were excluded from all further statistical analyses.

The inter‐rater agreement for PHOMS was 97.9%, with a kappa of 0.951. The proportion of PHOMS was significantly higher in patients with MS (16%) when compared to healthy control subjects (0%). Statistical significance increased further for comparison of the proportion of affected eyes (45 in MS and 0 in controls, *p <* 0.0001). There was no significant age difference between patients with MS who had PHOMS and those who did not (*p* = 0.54). There was no association between presence of PHOMS and the clinical disease course or disease duration (*p* = 0.26, *p* = 0.46, respectively).

Disease progression on the Expanded Disability Status Scale (EDSS) was mild (Table 1). There was no statistical relationship between progression on the EDSS and presence or absence of PHOMS (*p* = 0.23).

Management with disease‐modifying treatments (DMTs) was not associated with presence of PHOMS (chi‐squared test, *p* = 0.83). Of the 4 patients with fingolimod, only 1 had PHOMS.

**FIGURE 2:**
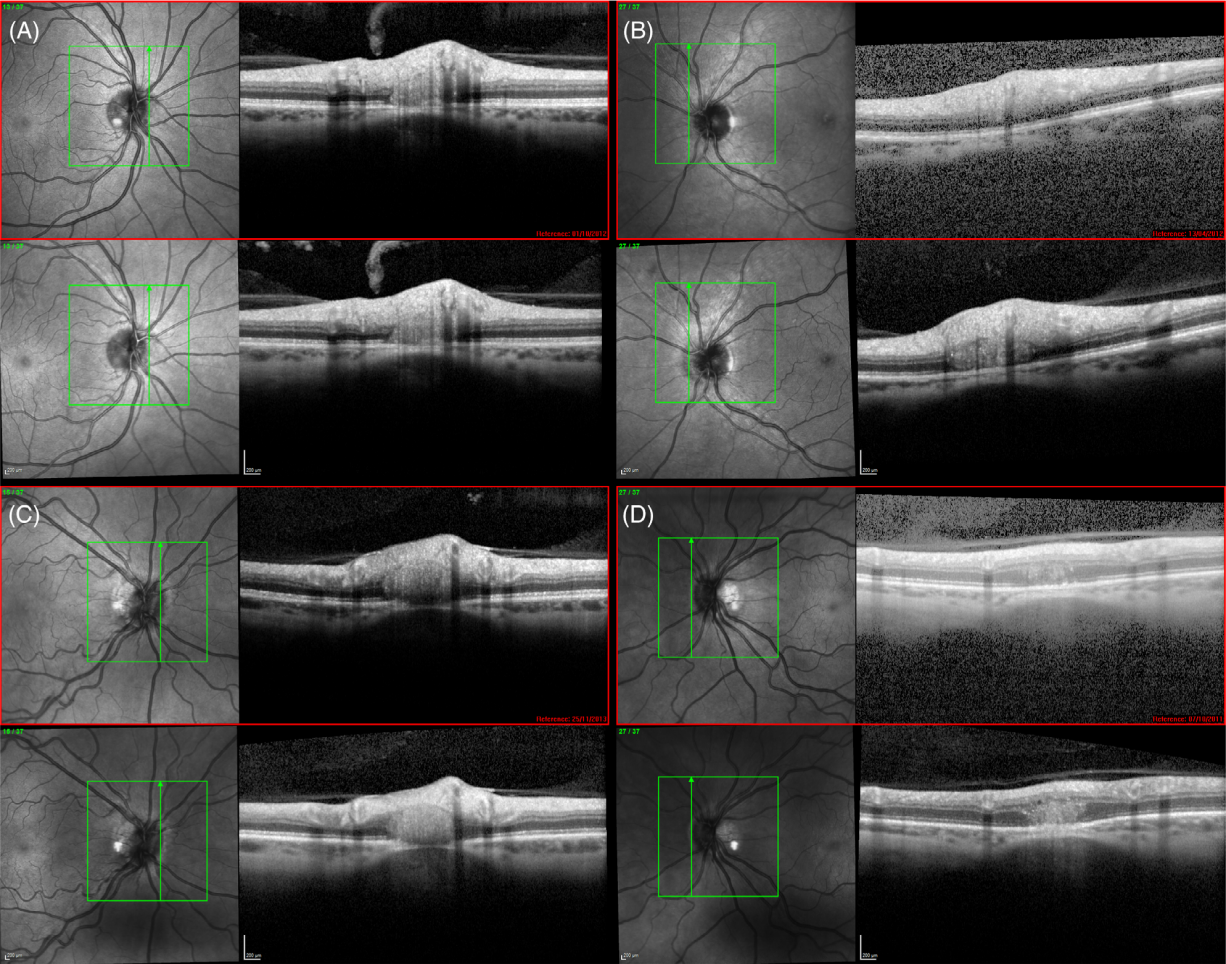
Examples of peripapillary hyper‐reflective ovoid masslike structures (PHOMS) in patients with multiple sclerosis. (A) A small PHOMS that remained stable in size over the 2‐year observation period. The baseline image is shown on the top (red frame) and the follow‐up image on the bottom. (B) A larger PHOMS that also remained stable over time. (C) A de novo PHOMS that developed. (D) A small PHOMS at baseline that has increased in size over 2 years. The confocal laser scanning ophthalmoscopy is shown on the left. The green lines indicate the location of the optical coherence tomographic B‐scan shown on the right. [Color figure can be viewed at www.annalsofneurology.org]

The longitudinal images demonstrated 3 patterns of PHOMS. First, PHOMS remained stable over the 2‐year observation period, as illustrated for a small PHOMS and a larger PHOMS (Fig 2). Second, there was de novo development of PHOMS. Finally, existing small PHOMS could increase in size.

### *PHOMS and MSON*

An episode of MSON had occurred in 144 eyes. The location was on the right in 74 and on the left in 70 eyes. Percentage of PHOMS in eyes affected by MSON was 10% on the right and 8% on the left. The percentage of PHOMS in eyes never affected by MSON was 14% on the right and 12% on the left. Overall, the presence of PHOMS was not related to a history of MSON (chi‐squared test, *p* > 0.05). The patterns of PHOMS (stable, de novo, increase) were not related to MSON.

### *PHOMS and Visual Pathway Integrity*

Table 2 summarizes data on visual pathway integrity in subjects with and without PHOMS.

**TABLE 2:** PHOMS and Visual Pathway Integrity in Multiple Sclerosis

	PHOMS−	PHOMS+	*p*
Eyes^a^			
n	427	40	
pRNFL baseline, μm	86.34 ± 13.91	84.83 ± 12.96	ns
pRNFL follow–up, μm	84.03 ± 14.44	84.57 ± 12.48	ns
Δ pRNFL, μm	−0.77 ± 1.98	−0.09 ± 2.22	ns
GCIPL baseline, μm	80.07 ± 14.58	79.53 ± 13.49	ns
GCIPL follow–up, μm	78.64 ± 14.92	78.06 ± 13.49	ns
Δ GCIPL, μm	−0.65 ± 1.51	−0.15 ± 1.19	ns
Patients^b^			
n	173	33	
Optic radiations			
MD	1.905 ± 0.197	1.863 ± 0.169	ns
FA	0.814 ± 0.084	0.845 ± 0.073	0.03^c^
Visual cortex			
V1	3.56 ± 0.25	3.52 ± 0.21	ns
V2	4.10 ± 0.23	4.06 ± 0.23	ns

a There were n = 5 eyes of patients with PHOMS and n = 33 eyes of patients without PHOMS who failed quality control for quantitative data for either the pRNFL or GCIPL.

b The magnetic resonance imaging metrics were not available from 1 patient with PHOMS and n = 49 patients without PHOMS.

c The Bonferroni‐adjusted *p* value for multiple comparisons (n = 4) is 0.0125.

FA = fractional anisotropy; GCIPL = ganglion cell inner plexiform layer; MD = mean diffusivity; ns = not significant; PHOMS = peripapillary hyper‐reflective ovoid masslike structure; pRNFL = peripapillary retinal nerve fiber layer.

In all eyes, there was progressive atrophy of the pRNFL and GCIPL over the 2‐year observation period. At baseline, eyes from patients with PHOMS did have a mildly thinner pRNFL and GCIPL if compared to patients without PHOMS, but this did not reach statistical significance. Progression of atrophy was more marked in eyes without PHOMS compared to eyes with PHOMS for both layers, again without reaching statistical significance.

In patients without PHOMS, the fractional anisotropy (FA) of the optic radiations was lower compared to patients with PHOMS, and this appeared to be statistically significant (*p* = 0.0363). This difference was no longer statistically significant after Bonferroni correction for multiple comparisons. Consistent with this finding, the mean diffusivity was higher in the optic radiations of patients with PHOMS compared to patients without PHOMS, but this did not reach statistical significance. Likewise, there was no statistically significant difference in the degree of atrophy of the occipital cortex either for V1 or V2 comparing the 2 groups (Table 2).

The patterns of PHOMS (stable, de novo, increase) were not related to these visual pathway data.

### *PHOMS in Non‐MS Disease Controls*

An additional retrospective case note review was performed on 267 patients who had for their routine clinical workup OCT optic nerve head volume imaging. Table 3 summarizes the subject characteristics for the disease groups. Overall, PHOMS were more frequent than ODD. Conditions associated most frequently with PHOMS were IIH (62%), ODD (47%), and anomalous optic discs (44%). The percentage of PHOMS in patients with an isolated optic neuritis (19%) and optic atrophy (12%) was comparable to the patients with MSON (18% of MSON eyes; see above).

**TABLE 3:** Subject Characteristics of the Retrospective Non–Multiple Sclerosis Disease Control Cohort (n = 267)

	ION	OA	Disc	Cog	Opt	Pain	IIH	MR	ODD	TMVL
Subjects	16	49	81	4	17	19	13	20	38	10
Eyes	32	98	162	8	34	38	26	40	76	20
Gender, F:M	10:6	22:27	53:28	2:2	12:5	15:4	12:1	9:11	21:17	6:4
Age, yr	44.5 (19.1)	53.2 (16.6)	34.0 (14.4)	40.8 (7.7)	38.4 (11.9)	36.0 (13.1)	29.7 (9.2)	44.7 (18.4)	38.4 (15.7)	43.5 (15.1)
ODD	1 (6%)	3 (6%)	6 (7%)	0 (0%)	0 (0%)	0 (0%)	0 (0%)	0 (0%)	38 (100%)	0 (0%)
PHOMS	3 (19%)	6 (12%)	36 (44%)	1 (1%)	0 (0%)	3 (16%)	8 (62%)	1 (1%)	18 (47%)	1 (10%)

Mean (SD) or n (%) is shown.

Cog = cognitive; Disc = anomalous discs; F = female; IIH = increased intracranial hypertension; ION = isolated optic neuritis; M = male; MR = medical retinal disease; OA = optic atrophy; ODD = isolated optic disc drusen; Opt = entoptic phenomena; Pain = headaches not due to IIH; PHOMS = peripapillary hyper‐reflective ovoid masslike structure; TMVL = nonembolic transient monocular visual field loss.

## Discussion

**FIGURE 3:**
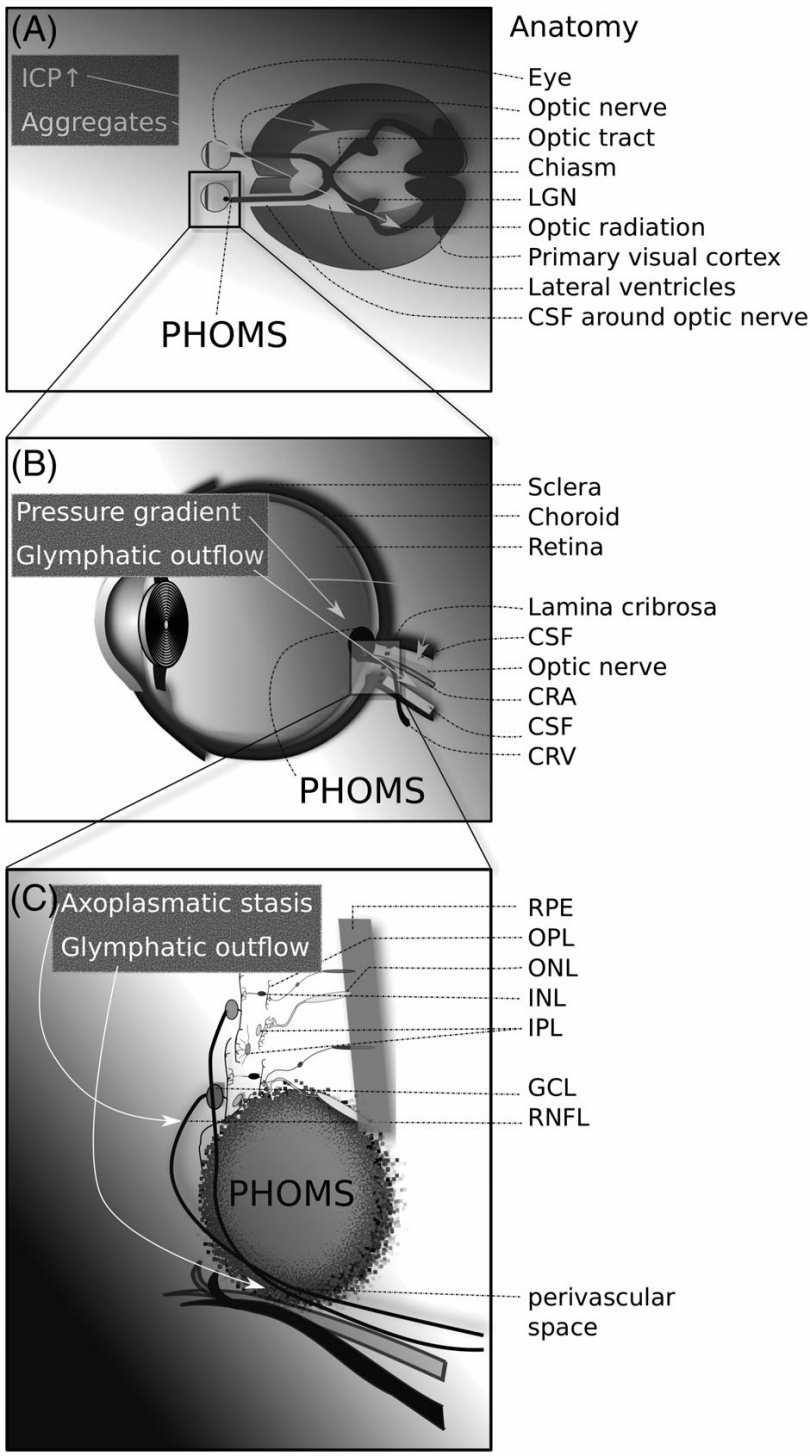
Development of peripapillary hyper‐reflective ovoid masslike structures (PHOMS) in multiple sclerosis. (A) The anatomy of the visual pathways in relation to the brain and cerebrospinal fluid (CSF) spaces. Increased intracranial pressure (ICP) and aggregate formation in the optic pathways may result in development of PHOMS (*arrows*). (B) PHOMS are located in the peripapillary area, where they are in close anatomical proximity to axons leaving the eye to form the optic nerve, the CSF in the optic nerve sheet, the central retinal artery (CRA), and the central retinal vein (CRV). A change of the translaminar pressure gradient (before/after lamina cribrosa) may result in axoplasmic stasis and reduced glymphatic outflow (*arrows*), resulting in PHOMS. (C) PHOMS have a local mass effect that typically displaces content from several retinal layers. This can give the impression of pseudopapilledema. Axoplasmic stasis and impaired glymphatic outflow through the perivascular space of the optic nerve may contribute to buildup of PHOMS (*arrows*). GCL = ganglion cell layer; INL = inner nuclear layer; IPL = inner plexiform layer; LGN = lateral geniculate ganglion; ONL = outer nuclear layer; OPL = outer plexiform layer; RNFL = retinal nerve fiber layer; RPE = retinal pigment epithelium.

There are 4 main findings from this study. First, PHOMS can clearly be seen in patients with MS using a routine optic nerve head OCT volume scan, with an excellent inter‐rater kappa of 0.951. Second, PHOMS are significantly more frequently present in patients with MS compared to healthy controls. Third, in patients with PHOMS, the optic radiations have a significantly higher FA compared to patients without PHOMS. Both findings are relevant because they provide indirect evidence for impaired axoplasmic flow in the visual pathways of patients with MS (Fig 3). Finally, there are 3 types of PHOMS: (1) PHOMS that remain stable, (2) PHOMS that increase in size, and (3) PHOMS that develop de novo.

It is important to note that presence of PHOMS is an observation independent of biases by key clinical features. This makes PHOMS an interesting new object to study and a hitherto unknown aspect of pathology in MS. Specifically, it has been excluded that PHOMS were observed more frequently in patients with MSON as compared to those who never experienced MSON.^10^ This observation was confirmed in the non‐MS cohort for isolated optic neuritis. This is relevant because PHOMS can be observed in the course of a whole range of etiology leading to optic disc swelling (personal observation, A.P.). Next, PHOMS were not related to the degree of either pRNFL or GCIPL atrophy. Neither were degree of atrophy of the pRNFL or GCIPL associated with PHOMS. Longitudinally, PHOMS, even if developing, were not related to progression of atrophy. Again, the finding was confirmed by the data from the non‐MS cohort for optic atrophy. This is an observation that, for example, can be made with ODD causing progressive visual field defects.^18^ The known association between more severe retinal inner layer atrophy and progression of disability on the EDSS^19^ cannot be shown for PHOMS. There was no relationship between PHOMS and use of DMTs. This is relevant because, for example, fingolimod has been identified as a cause for macular edema.^20^ Likewise, there was no association of PHOMS with demographic data or disease duration. The latter can, however, also be interpreted as a limitation of the study, because we cannot comment on PHOMS during the early disease course of MS. Patients in this study had a long disease duration of about 20 years. A strength of the study is, however, the longitudinal data, with an averaged follow‐up period of 26 to 27 months.

After having ruled out an association of PHOMS with demographic or clinical data, it is possible to return to the observation with an unbiased mind. In MS, there are very few patients with PHOMS, and in these patients, one can observe a significant increase of the FA in the optic radiations. Anatomically, the first observation locates to the anterior and the second to the posterior optic pathways. What could be an explanation connecting the two?

There are at least 3 potential explanations to discuss: (1) axoplasmic stasis and localized aggregate formation, (2) the glymphatic system, and (3) the translaminar pressure gradient at the optic disc.

The first argument builds on an earlier indirect observation of impaired axonal transport and aggregated formation.^21^ In MS, some axons, particularly those adjacent to MS lesions, show signs of increased neurofilament compactness and aggregation in the axolemma. Neurofilaments are a key component of the axonal cytoskeleton.^22^, ^23^ Therefore, the observation of axonal swellings indicating a reversible form of axonal damage is intriguing.^24^ The images shown in this elegant study demonstrate local accumulation of neurofilament proteins in axonal swelling.^24^ Could PHOMS be a late sign for reversible axonal damage in MS? Future immunohistochemical postmortem studies of PHOMS in the retina of patients with MS will be needed to clarify whether neurofilament proteins or myelin products can be found in PHOMS. Such future studies should also investigate the role of phosphorylation of neurofilaments and other proteins, which significantly affect MRI metrics.^25^ One question arising from the observation of the increased FA is whether proton mobility in the optic pathways (see Fig 3A) of patients with PHOMS is influenced by accumulation, aggregation, and phosphorylation of proteins, all contributing to impaired axonal flow. This line of argumentation may be useful for helping to explain the otherwise paradoxical observation of an increase of the RNFL over time in some patients with MS and in experimental models.^26^, ^27^

The second hypothesis builds on the observation of impairment of the glymphatic system.^28^ Hamann et al have demonstrated that water channel (aquaporin) distribution in the eye provides an excellent anatomical basis for a presumed ocular glymphatic system.^29^, ^30^ Therefore, it could be possible that an impaired glymphatic system through aquaporins in MS reduces the ability to remove extracellular waste products such as compounds extravasated during axoplasmic stasis (see Fig 3C).^31^ There have been independent lines of argumentation in the past 5 years raising the likelihood of such a retinal glymphatic system.^32^, ^33^, ^34^, ^35^ The longitudinal data from this study show that the increase of size of PHOMS had been very mild over a 2‐year period. Such a slow dynamic would be more consistent with a glymphatic problem, rather than being a consequence of more acute pathology, particularly after relevant demographic and clinical factors have been ruled out.

The third hypothesis is related to the second and offers a mechanistic approach to the previous two. The translaminar pressure gradient at the optic disc (see Fig 3B) has been suggested as a relevant factor in driving neurodegeneration in a chronic optic neuropathy, glaucoma.^36^ We are not aware of data on PHOMS in the glaucoma literature, but another example of transient change of the translaminar pressure gradient at the optic disc is IIH. The development of PHOMS in patients with IIH has been observed, as well as PHOMS regression after treatment^37^ (personal observation, A.P.). Recent reports on an elevated lumbar opening pressure in MS come from the pediatric literature.^38^, ^39^ However, the observation had been made reliably by experienced neuro‐ophthalmologists anecdotally in adults.^40^ All of their 3 patients reported headaches, and lumbar puncture opening cerebrospinal fluid pressures were *>*29cm H_2_O, 42cm H_2_O, and 25cm H_2_O.^40^ Contemporary routine examination of the cerebrospinal fluid in patients with MS does not include measurement of the opening pressure.^41^ Future studies investigating the hypothesis that there could be intermittent intracranial pressure elevation in MS (see Fig 3B) are advised to follow a well‐designed protocol for calibrated pressure measurements of the eye and brain.^42^ This will be of particular interest in patients in whom PHOMS develop de novo or increase in size over time. These observations provide indirect evidence for a glymphatic system that connects the eye with the brain.

Above interpretations of our data also highlight the most relevant shortcomings. There are no recent histological data showing PHOMS in MS that shed light on the underlying pathology. The published immunohistochemical images of postmortem optic discs in MS by Green et al show degrees of axonal atrophy, but these samples did not contain eyes from patients with PHOMS.^43^ Arguably, the 3 cases presented historically by Norris did not present optic neuritis as defined by von Graefe and Nettleship.^1^, ^2^ Instead, he described optic disc edema in the context of other pathology.^3^ Nevertheless, these authors reported an important histological observation that, given the alternative pathology and the findings in our non‐MS cohort, remind us that PHOMS are not specific to MS. Similar to what has been reported for other new OCT‐based observations in MS, PHOMS can be found with a whole range of clinical pathologies, and in a clinical context are most frequently misinterpreted as pseudopapilledema (personal observation, A.P.).

Technical limitations of the study are related to software updates. Very small ODD below Bruch's membrane can escape detection without use of EDI. The ODD consortium has therefore developed a highly sensitive ODD imaging protocol that should be employed in future studies on PHOMS in MS (see Table 1 in Malmqvist et al^5^). Likewise, we did not quantitatively assess the relative afferent pupillary defect, which would be an interesting additional metric for visual function to be correlated to PHOMS.^44^, ^45^

Clinical limitations of the study are related to the retrospective nature of the non‐MS cohort. The retrospective cohort does, however, permit examining in more general terms the association of PHOMS with other diseases. This is clinically relevant because of the differential diagnosis of IIH. If PHOMS are misinterpreted clinically as true disc swelling, then there is a risk of overestimating IIH. In 16% of cases with a primary headache disorder, the presence of PHOMS will give the impression of pseudopapilledema. The difficulty interpreting PHOMS as a cause for pseudopapilledema is also reflected in the high referral pattern to clinics of patients with what has been classified as an “anomalous disc.” In 44%, this was due to PHOMS. The presence of PHOMS in 19% of patients with an isolated optic neuropathy and 12% of patients with optic atrophy will require future research. For example, the ODD Consortium has identified PHOMS as a novel, independent risk factor for young onset (*<*50 years), nonarteritic ischemic optic neuropathy. Likewise, almost half of all patients with ODD also harbor PHOMS. Taken together, the clinical limitations of the post hoc retrospective addition of the non‐MS cohort does still add valuable clinical information.

It is worthwhile to reflect on the study limitations in a broader context. To date, work on the glymphatic system largely relies on histological data from tracer studies in rodents.^46^, ^47^ There is a need for other methodological approaches suitable for longitudinal in vivo human studies. This is required to study any presumed relationships to disease processes. The hypothesis to be investigated further in MS research is whether impairment of the glymphatic system could contribute to explaining reduced clearance of potentially immunogenic compounds from the paravascular space. Approaches in this direction are needed to explain the pathognomonic but enigmatic perivascular compartmentalization of lymphocytes in the brain of patients with MS.^48^ A further limitation is that the only MRI parameter we found to be associated with presence of PHOMS, FA, does not have pathological specificity. Lower FA is typically thought to represent bundle atrophy. FA can increase as a result of restricted perpendicular diffusivity, facilitated parallel diffusivity, or some combination of the two. Future studies may benefit from advanced multimodal brain imaging, including positron emission tomography with novel dynamic tracers.^49^ Other limitations of our study relate to the regular update of consensus criteria. For internal consistency with our previous publications,^12^, ^13^ we adhered to the 2010 revision of the McDonald criteria and the 1996 Lublins classification. All of our patients also met the 2017 revision of the McDonald criteria.^41^ However, there have been relevant changes to the disease course in the revision to the disease course.^50^ The main difference relates to "active" and "nonactive" disease. In this context, observed development of PHOMS over time may be interrogated as an alternative approach to recognize disease activity that may be of interest for future revisions of such classifications. Probably the most relevant limitation comes, however, from a recent debate.^51^, ^52^, ^53^, ^54^ The existence of a glymphatic transport system from the eye to the brain has just been demonstrated experimentally in rodents.^55^

In conclusion, this study shows that a small proportion of patients with MS harbor PHOMS. In these patients, PHOMS can slowly increase in size over time or develop de novo. There are plausible mechanisms that can explain this development. The presence of PHOMS may be caused by axoplasmic stasis, impairment of a presumed glymphatic system from the eye through the optic nerve, or change of the translaminar pressure gradient at the optic disc. Taken together, PHOMS are a novel finding in MS that might be useful for study of a hitherto unexplored pathway in MS, the glymphatic system.

## Author Contributions

A.P., B.M.J.U., and L.J.B. contributed to the conception and design of the study; A.P., D.C., D.P.C., L.J.B., A.K.D., P.A.K and S.H. contributed to the acquisition and analysis of data; A.P. drafted the text and prepared the figures.

## Potential Conflicts of Interest

A.P. is part of the steering committee of the ANGI network, which is sponsored by Zeiss, and steering committee of the OCTiMS study, which is sponsored by Novartis and reports speaker fees from Heidelberg Engineering. P.A.K. reports speaker fees from Zeiss, Topcon, Heidelberg Engineering, and Haag‐Streit and has received consultancy fees from DeepMind and Optos. The other authors have nothing to report.
